# Union Efforts to Reduce COVID-19 Infections Among Grocery Store Workers

**DOI:** 10.1177/10482911211015676

**Published:** 2021-05-08

**Authors:** Nancy A. Crowell, Alan Hanson, Louisa Boudreau, Robyn Robbins, Rosemary K. Sokas

**Affiliations:** 1 School of Nursing and Health Studies, Georgetown University, Washington, DC, USA; 2 United Food and Commercial Workers Union

**Keywords:** grocery workers, occupational safety and health, unions, worker protection, COVID-19

## Abstract

Grocery store workers are essential workers, but often have not been provided with appropriate protection during the current pandemic. This report describes efforts made by one union local to protect workers, including negotiated paid sick leave and specific safety practices. Union representatives from 319 stores completed 1612 in-store surveys to assess compliance between 23 April 2020 and 31 August 2020. Employers provided the union with lists of workers confirmed to have COVID-19 infection through 31 December 2020. Worker infection rates were calculated using store employees represented by the union as the denominator and compared to cumulative county infection rates; outcome was dichotomized as rates higher or lower than background rates. Restrictions on reusable bags and management enforcement of customer mask usage were most strongly associated with COVID-19 rates lower than rates in the surrounding county. Stores that responded positively to worker complaints also had better outcomes. The union is currently engaging to promote improved ventilation and vaccination uptake.

## Introduction

Grocery and supermarket workers are indispensable for community survival. Their jobs require physical exertion and customer contact. They experience higher rates of nonfatal injuries compared to the U.S. average^1^ and, as public-facing workers, have had increased or borderline increased rates of acute respiratory illness or influenza-like illnesses (ILI) documented in the past.^2^ They are considered essential workers whose jobs place them at risk for exposure, yet they are low-wage workers who often lack the safety measures required to prevent the occupational transmission of SARS CoV-2, the virus causing the COVID-19 pandemic. Early in the current pandemic, an investigation of COVID-19 rates in a Massachusetts grocery store found that of the 104 workers screened by PCR testing, 20 percent had positive viral assays; fewer than half of the infected workers were symptomatic.^3^ Testing occurred in May, prior to a state-wide mask mandate (personal communication). A previous study conducted during the H1N1 pandemic surveyed a nationally representative sample of 2079 American adults and found increased levels of ILI among those who had no paid sick leave or who would be unable to afford to stay home for seven to ten days.^4^

Although mask usage is not considered a form of respiratory protection, there has been growing evidence that it is useful both as a form of source control and to provide some level of personal protection. Twenty-four Kansas counties that opted into a mask mandate as of 3 July 2020 were compared to eighty-one counties that opted out; pre-mandate COVID-19 incidence rates were increasing at a similar rate across all counties. Postmandate seven-day rolling average rates decreased 6 percent in counties with mask ordinances (mean decrease = 0.08 cases per 100,000 per day; 95 percent confidence interval [CI] = –0.14 to –0.03), but rose by 100 percent in counties that did not enact any mask requirements (mean increase = 0.11 cases per 100,000 per day; 95 percent CI = 0.01–0.21).^5^

This report was developed in response to a request from the United Food and Commercial Workers (UFCW) Union, Local 400, to review store safety surveys collected by union representatives, and to evaluate COVID-19 illness information among workers represented by the union as reported by stores under contract.

## UFCW Local 400

UFCW Local 400 represents more than 35,000 workers from various businesses in the Mid-Atlantic Region (District of Columbia, Maryland, Virginia, West Virginia, Kentucky, Tennessee, and Ohio), of whom 26,000 work in grocery store chains. Early in the pandemic, the union developed a series of safety measures that were negotiated with store chain management, and further negotiated fourteen days of paid leave for workers with known or suspected COVID-19 exposure or infection. The union worked with shop stewards and employees serving on store-level safety and health committees to conduct store surveys and checklists to determine store compliance with negotiated safety measures and, beginning on 23 April 2020, the local union contacted representatives or other members to collect survey results. A union contract affords employees the opportunity to raise concerns with management without fear of retaliation. The safety survey asked if employees had raised safety concerns with management and, if so, how management addressed them. Union leadership compiled the information into an Excel spreadsheet reporting store identification and zip code.

## Georgetown University Partnership Engagement

Georgetown University and UFCW Local and International representatives met iteratively to identify questions of interest, outlined below:

1. What was the distribution of the safety measures, both individual ones and a summary score?
2. How did this change over time or by location?
3. If workers identified problems and raised them with management, did the store safety score change in the subsequent survey?
4. How did the rates of infections among grocery store workers compare to the background rates for the county, and did these vary by safety measures or by state and local measures?

## Methods

The Georgetown University Institutional Review Board reviewed the project proposal to ensure human subjects protection and granted exempt status based on the use of previously collected anonymous data. Georgetown partners obtained spreadsheets, cleaned data, and entered data into STATA 16 and SPSS 26 for further evaluation. Additional state and county information was obtained for various pandemic-related mandates, but because of the numbers of changes, the focus was placed on mask orders. Background county rates of COVID-19 infections were obtained from sources described below and expressed as rates per county population.

### Safety Scores

There were originally twenty-two safety items on the survey. The items “Aisles closed for restocking” and “Perimeter departments closed for restocking,” and the items “Self-checkout operating every other register” and “Self-checkout closed” were infrequently implemented and were therefore combined, resulting in the following twenty items:

- Social distancing being practiced
- Enforcing customer shopping limits
- One entrance closed or used for exit only
- One-way aisles
- Aisles closed for restocking and/or perimeter departments closed for restocking
- Social distancing floor signage
- Customers required to wear masks
- Management enforcing customer mask wearing requirement
- Hand washing every thirty minutes
- Hand sanitizer available at work stations
- Work stations cleaned every thirty minutes
- Breakroom social distancing
- Employees required to wear masks
- Masks/gloves/sanitizer provided by company
- Training on proper putting on/taking off masks
- Frequently touched surfaces cleaned regularly
- Operating every other check stand
- Self-checkout operating every other register and/or self-checkout closed
- Reusable bags banned or cashiers/baggers not required to handle reusable bags
- Social distancing while waiting in line

A “yes” on an item was counted as one point while a “no” was counted as zero. A total score was obtained by summing the points to all twenty items. Fifty-four surveys that were submitted via the Google spreadsheet to UFCW were duplicates and eliminated from this analysis. Surveys were considered duplications if they were submitted by the same person within two days of each other with identical answers. The number of surveys submitted by stores ranged from only one to twelve. In order to not give undue influence to stores who submitted a greater number of surveys, all percentages and means have been adjusted to account for the number of surveys submitted by each store in a month by averaging the responses for all surveys from each store in each month.

Following the American Statistical Association (ASA) Statement on Statistical Significance and *p* values,^6^ we are not using the term “statistical significance” based on *p* values. We report *p* values associated with statistical tests, but recognize that while a *p* value indicates the probability that the difference in observed means or proportions would have been this large or larger if there were no real differences between them, it tells very little about the size of the difference. The size of the difference is better described by an effect size, such as Cohen’s *d*, which is the amount of standard deviation difference in the means. The ASA also recommends providing a measure of the probability that the results were due to chance. One such measure is the false positive risk (FPR)^7,8^ that is reported as well. A combination of a relatively small *p* value, relatively large effect size, and relatively small *FPR*, suggests the items that best differentiated stores that were above county COVID-19 rates from other stores.

Store management provided Local 400 with a list of all employees represented by the union confirmed to have tested positive for COVID-19 and the date each infected individual last worked. The union removed all personally identifiable information and sent the numbers to Georgetown partners with date last worked and store ID, as well as the total number of employees the union represented in that store (the phrase “employee represented by the union” is used in place of “union member” since workers in right-to-work states may not be union members but would still have union protections). No public health or other testing was ever conducted onsite at any of the stores, and no demographic information (age, race, ethnicity, gender) was available either for infected workers or the total number of workers employed. Worker infection rates per store were calculated at the last day of the month from March 2020 through November 2020.

Worker infection rates differed by factors such as demographics, for which we could not control. Rather than comparing worker infection rates from stores in different counties, the comparison of in-store worker infection rates to county rates served as a measure of the county’s background contribution to the store’s infection rate. To examine whether safety practices reported by stores in the safety surveys affected COVID-19 rates, we compared the percentage of time that stores engaged in each safety practice between stores in which worker infection rates exceeded the background county rate and those in which the worker infection rate fell below the county rate. Between April and August 30, 2020, stores submitted surveys periodically. For each store, answers for all surveys submitted were averaged to produce an overall proportion of the time each safety practice was reported. Because of the variability in store survey completion, we used the dichotomous outcome of infection rates above or not above county background rates as the marker for negative or positive outcomes rather than store safety scores. Because no store exactly matched the county background rate, they are reported as above or below.

County COVID-19 cumulative cases were obtained from the *New York Times* database (available to download from https://github.com/nytimes/covid-19-data). The cumulative case number on each date was divided by the county population obtained from the Johns Hopkins COVID-19 tracking web site (https://coronavirus.jhu.edu/us-map), whose source is the American Community Survey of the U.S. Census Bureau.

Open-ended comments were reviewed separately by two investigators to assess whether the union representative reported raising an issue with store management and whether the outcome was positive, negative, or not able to be determined. Differences were reconciled through discussion and the consensus determination was entered into the data.

## Results

A total of 319 stores had safety survey data reported between April and August 31, 2020. An additional seven stores reported COVID-19 cases, but had not completed any safety surveys so are not included in the main analyses. Table 1 describes the 319 stores by region.

**Table 1.** Stores by Region.

	n	%
DC	22	6.9
Maryland	93	29.2
Northern Virginia	92	28.8
Rest of Virginia	68	21.3
West Virginia	35	11.0
KY, OH, TN	9	2.8

*Note*. DC = District of Columbia; KY = Kentucky; OH = Ohio; TN = Tennessee.

**Figure 1.**
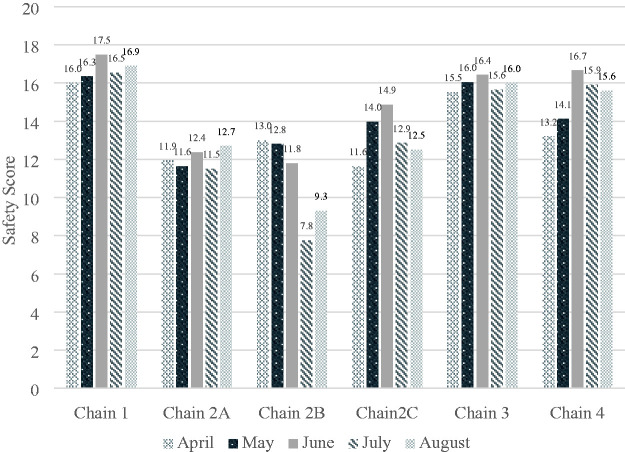
Mean safety score by chain, adjusted by number of responses by store in each month.

A total of 1612 store surveys were completed between 23 April 2020 and 31 August 2020. Store safety scores differed by month and by store chain. Figure 1 displays the monthly averages by grocery store chain through August. Reporting a safety violation appeared to make little difference on the subsequent safety score. The subsequent safety score increased in 37 percent of the stores after a safety violation was reported, but also increased in 30 percent of the stores without a reported safety violation (*p *=* *.38). There were no differences in changes in safety scores based on the management response to the reported safety violation.

A total of ninety-six of the 319 stores (30.1%) experienced a COVID-19 outbreak, that is, three or more cases within a fourteen-day period. Table 2 shows the number of stores with outbreaks by store chain. There was a difference in number of outbreaks by store chain, χ^2^(5) = 22.7, *p *<* *.001, Cramer’s *V *=* *.25, *FPR *=* *0.006. Note that Chain 2 reports results by three separate regions, representing three separate bargaining units, labeled A, B, and C in Table 2. Chain 2 had a higher percentage of stores with outbreaks in all regions.

**Table 2.** Percentage of Stores in Chain with Outbreaks.

Employer	Number of stores in chain with outbreaks
	n (%)
Chain 1	33 (26.8)
Chain 3	15 (17.2)
Chain 4	1 (7.1)
Chain 2 A	12 (48.0)
Chain 2B	18 (46.1)
Chain 2 C	17 (42.5)

Outbreaks varied by month, with November and December having the highest number of outbreaks (see Table 3). This reflects the spread of the pandemic to these stores, which are largely in the Mid-Atlantic region of the United States. Note that the total number of stores is greater in Table 3 than in Table 2 because some stores had outbreaks in more than one month.

**Table 3.** Number of Stores with Outbreaks by Month.

Month	Number of Stores with Outbreaks
March	1
April	12
May	11
June	2
July	3
August	2
September	1
October	11
November	25
December	54

Cumulative store COVID-19 rates were compared to the rate in the county in which the store is located on September 30, October 31, and November 30, 2020. There were differences in percentages of stores with higher than background rates by region in September, October, and November. In September, District of Columbia and Northern Virginia had a higher percentage of stores above the background rate than other regions. In October and November, Maryland and the rest of Virginia had a significantly lower percentage of stores above the background rate than other regions; the percentages in West Virginia and Kentucky/Ohio/Tennessee surpassed all other regions in October and November. It should be noted that there were relatively few stores in the Kentucky/Ohio/Tennessee group.

Table 4 shows the comparison of average rates of compliance from April through August 2020 for each safety practice between stores with worker rates of COVID-19 above the background county rate and stores whose worker COVID 19 infection rates were below county rates as of the end of October 2020. A similar analysis was completed for rates at the end of September and November (available upon request from first author and online in Supplemental Files). October was selected for presentation in Table 4, as it provided adequate time after the last safety survey for effects on COVID-19 rates to manifest.

**Table 4.** Mean Percentage of Time Between April and August 2020 That Stores Practiced Specific Safety Behaviors by Whether or Not Store COVID-19 Rate Was Above County Rate or Not in October 2020.

Above county rate	Above	Below			
	%	%	*p*	*d*	FPR
Reusable bags banned or cashiers/baggers not required to handle	92.4	96.7	.004	.35	.03
Management enforcing customer mask wearing	42.4	54.8	.02	.29	.10
customers required to wear masks	63.5	72.9	.02	.28	.11
Frequently touched surfaces cleaned regularly	85.5	90.2	.09	.20	.32
Masks/gloves/sanitizer (PPE) provided by company	98.3	99.4	.13	.18	.39
Work stations cleaned every 30 minutes	84.0	87.8	.21	.15	.48
One-way aisles	79.8	74.4	.26	−.14	.51
Social distancing being practiced	85.1	88.6	.27	.13	.52
Social distancing while waiting in line	94.1	95.6	.29	.13	.53
Breakroom social distancing	69.9	73.6	.38	.11	.58
Operating every other check stand	65.8	69.3	.38	.11	.58
Employees required to wear masks	97.2	98.0	.61	.06	.64
Aisles or peripheral departments closed for restocking	13.1	11.8	.67	−.05	.65
Hand washing every 30 minutes	75.5	73.8	.69	−.05	.65
Self service checkouts closed or operating every other station	45.8	43.1	.54	−.03	.64
Hand sanitizer available at work stations	98.0	98.2	.82	.03	.66
One entrance closed or used for exit only	70.4	71.0	.86	.02	.66
Training on proper putting on/taking off of masks	66.3	65.6	.90	−.02	.67
Social distancing floor signs	96.0	96.1	.96	.01	.67
customer shopping limits	71.1	71.0	.99	−.001	.67

*Note*. FPR = false positive risk; PPE = personal protective equipment.

There was a tendency for stores with worker COVID-19 infection rates higher than the surrounding county to implement safety measures less often than stores with worker infection rates below the county rate; however, these differences were small. Based on *p* value, effect size, and *FPR*, the following items best differentiated stores with worker COVID-19 rates above the background county rate from stores with worker rates below county rates: reusable bags banned or cashiers/baggers not required to handle, management enforcing customer mask requirements, customers required to wear masks, and frequently touched surfaces cleaned regularly. Restrictions on reusable bags showed consistent effects on infection rates from September (*p *=* *.08, *d *=* *.22, *FPR *=* *.29) through November (*p *=* *.005, *d *=* *.34, *FPR *=* *.04). Practices that differentiated store rates in September but not October included social distancing being practiced (*p *=* *.04, *d *=* *.25, *FPR *=* *.20), work stations cleaned every thirty minutes (*p *=* *.08, *d *=* *.22, *FPR *=* *.29), and personal protective equipment provided by the company (*p *=* *.09, *d *=* *.21, *FPR *=* *.33). Some items that did not differentiate (such as employees wearing masks or having hand sanitizer) were in practice in nearly all stores all of the time. The use of one-way aisles, however, appeared not to be useful and potentially counterproductive.

As described earlier, the safety survey also asked whether or not safety concerns had been reported to the management. Workers who reported safety concerns to management were somewhat more likely to be from stores with COVID-19 rates above the background county rate than workers in stores in which no reports were made. The effect size was very small, however, (*d* between −.08 and −.04) and *FPR* high (between 62% and 65%). Stores in which worker concerns were met with positive responses from management were more likely to have worker COVID-19 infection rates below county rates compared to those stores in which management response was negative. Workers reporting concerns in stores with higher than background COVID-19 rates were more likely to get negative responses from management. The effect size for negative responses in September and October were .25 and .34, respectively, meaningful effects for social interventions (although statistically considered small). *FPR* in September was 46 percent and in October 27 percent.

**Figure 2.**
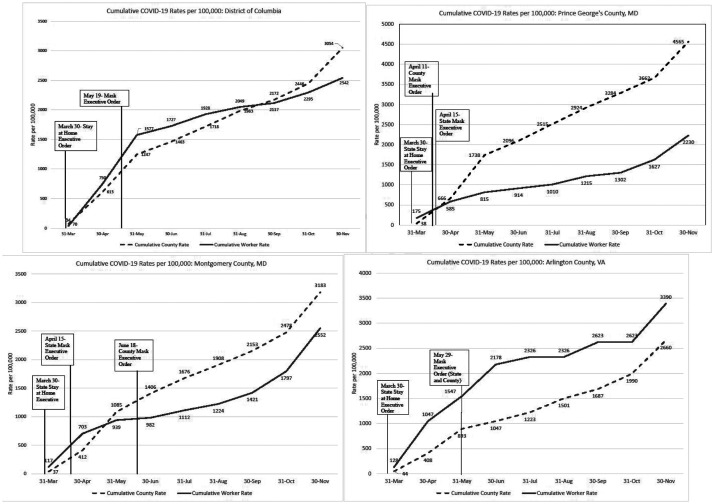
Comparison of worker (store) rates to county rates in selected counties.

Figure 2 illustrates the relationship between cumulative rates of COVID-19 infections among grocery workers and the background county rates in the largest city in the region and some of its surrounding counties, including three jurisdictions (the District of Columbia and parts of Maryland and Virginia). Local and state mask mandates are noted in each. This region experienced the earliest rise in COVID-19 cases among areas covered in this study, had the largest populations, and included the most stores and multiple store chains. Although the relationship to background county rates varied, in most instances the initial worker rates are higher, and precede the rise in background county rates. Local mask mandates appear to reduce the rate of increase of both worker and background rates in a number of instances. (Additional county figures are available in Online Supplemental Files.)

## Discussion

This report supports reported work in long-term care facilities that demonstrated the importance of unions in reducing adverse effects from the COVID-19 pandemic.^9^ In this study, the UFCW had made a number of important efforts to reduce the risk of infection to its members. First, it negotiated paid sick leave that provided workers with the ability to stay home following infection or close exposure without facing lost wages, encouraging symptom reporting and testing. Although some safety measures were universally applied and therefore difficult to assess for relative effectiveness, the safety surveys documented a wide range of compliance with some practices, among them management enforcement of customer mask usage. Our findings of improved outcomes among workers in stores in which customer mask use was enforced by management as well as the appearance of changes in the rate of both store and county rates following early mask mandates support previous findings.^5^

Restrictions on use of reusable bags were consistently associated with better outcomes. It is unclear whether restricting reusable bags is a marker for other behaviors, such as limiting social contact, or useful in itself. Social distancing and regular cleaning appeared to be useful as well, although the effect of management-required mask usage by customers was most strongly associated with better outcomes. On the other hand, our findings suggest that one-way aisles may not be helpful. Stores in which workers raised complaints performed well when management was perceived to have responded to those complaints. Note that none of the work sites in our report participated in comprehensive screening. Workers obtained testing for the same reasons those in the general population did, making it unlikely that testing bias accounted for the differences with the background county rates.

Limitations include reduced precision in outcome measures by dichotomizing positive and negative outcomes into those in which the workers rates were below or above the county level. We did not have worker information for those instances in which the worker may have commuted from a different county with different COVID-19 rates than those for the county of the store. Nor did we have information on other possible places of exposure for workers. In addition, we were unable to distinguish among workers conducting different tasks within the stores. Finally, we had no demographic information on workers.

This report supports the importance of union-negotiated safety and health measures during the pandemic and suggests the importance of labor–management cooperation. It is possible that the impact of management’s response to raised safety concerns is also reflected in the safety scores. Enforcing the customer mask mandate and banning the use of reusable bags require the active intervention of management, either by directly engaging with customers or by directing store security to do so. We believe programs training front-line managers to exert safety leadership, such as the Foundations for Safety Leadership developed for the construction industry (CPWR),^10^ should be explored for use in grocery and other industries.

Protecting grocery workers also has implications for addressing the disparities among communities of color. Although we did not have access to demographic information for infected workers, national data indicate racial, ethnic, and economic disparities. Grocery and retail cashiers, who represent fully 18.3 percent of front line workers nationally, are disproportionately female (71.8%), non-white (44.6%), and living below 200 percent of the federal poverty level (42.7%).^11^ The Advisory Committee for Immunization Practices and the Centers for Disease Control and Prevention have recommended grocery workers for prioritization in the second group for immunization, following healthcare workers and residents and staff in nursing homes. ^12^ However, these are recommendations only, and prioritization of vaccination efforts is determined at the state level. Concerns about vaccine hesitancy and disparate rates of vaccine administration among racial and ethnic minorities have been widely discussed. By engaging in-store workers as leaders and providing consistent, supportive information about the importance and safety of vaccines, the union has prepared its members for participation, although scarcity of vaccine supply has limited the outcome measure to anecdotal instances of high vaccine uptake in several stores.

The standard industrial hygiene approach to prevention features engineering controls that include infrastructure improvements in ventilation, such as the use of ultraviolet germicidal irradiation within air ducts. Currently available methods have been demonstrated to reduce risk, and new methods are under investigation.^13^ The union is actively engaged in negotiating ventilation concerns, particularly in stores experiencing outbreaks.

Finally, the escalation in cases and outbreaks in stores and counties highlights the critical importance of prioritizing grocery workers for COVID-19 vaccination. Instances of stores with pharmacies being crowded with vaccine-seeking customers emphasizes the need to fully immunize workers first and to maintain strict adherence to additional public health prevention measures, including management enforcement of customer mask usage.

## Supplemental Material

sj-pdf-1-new-10.1177_10482911211015676 - Supplemental material for Union Efforts to Reduce COVID-19 Infections Among Grocery Store Workers Click here for additional data file. Supplemental material, sj-pdf-1-new-10.1177_10482911211015676 for Union Efforts to Reduce COVID-19 Infections Among Grocery Store Workers by Nancy A. Crowell, Alan Hanson, Louisa Boudreau, Robyn Robbins and Rosemary K. Sokas in NEW SOLUTIONS: A Journal of Environmental and Occupational Health Policy
