# Supplementary material for: Union Efforts to Reduce COVID-19 Infections Among Grocery Store Workers
Source: New Solut. 2021 May 8;31(2):170–7. doi: 10.1177/10482911211015676 (PMC8107491; doi:10.1177/10482911211015676)
Supplement: sj-pdf-1-new-10.1177_10482911211015676 - Supplemental material for Union Efforts to Reduce COVID-19 Infections Among Grocery Store Workers [file sj-pdf-1-new-10.1177_10482911211015676.pdf]

Union Efforts to Reduce Covid-19 Infections among Grocery Store Workers Introduction

Nancy A. Crowell, Alan Hanson, Louisa Boudreau, Robyn Robbins, Rosemary K. Sokas

Supplemental Figures and Tables

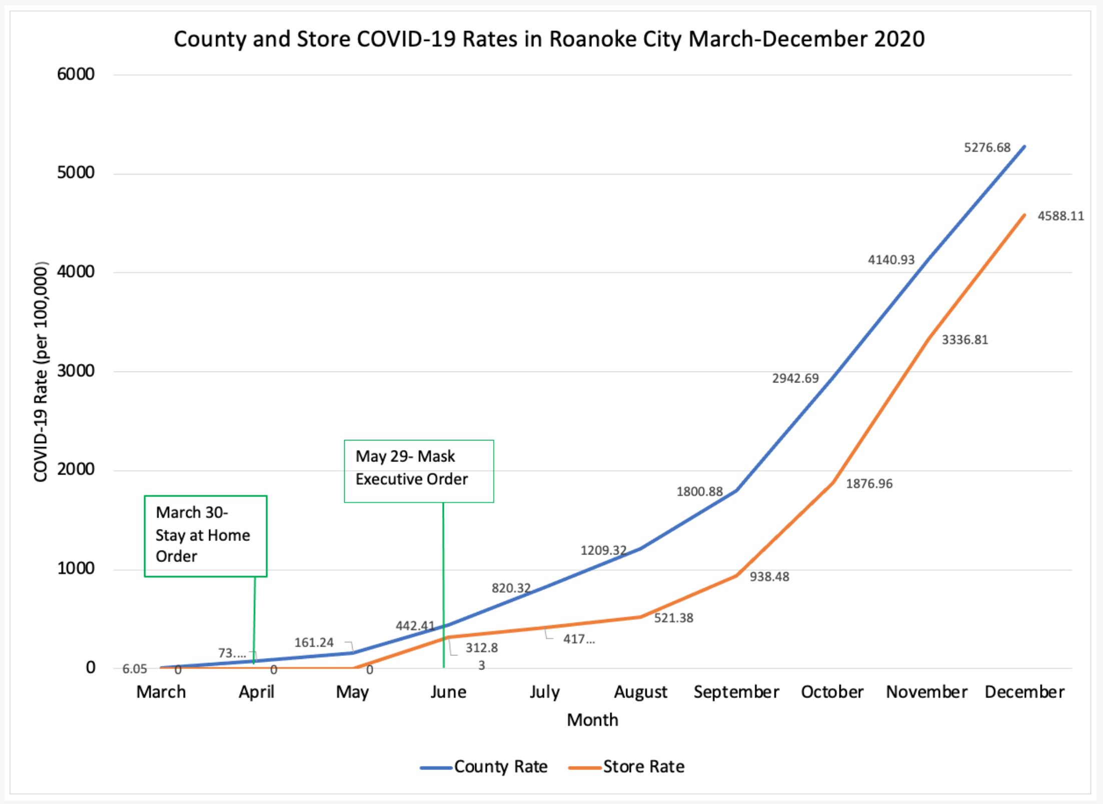

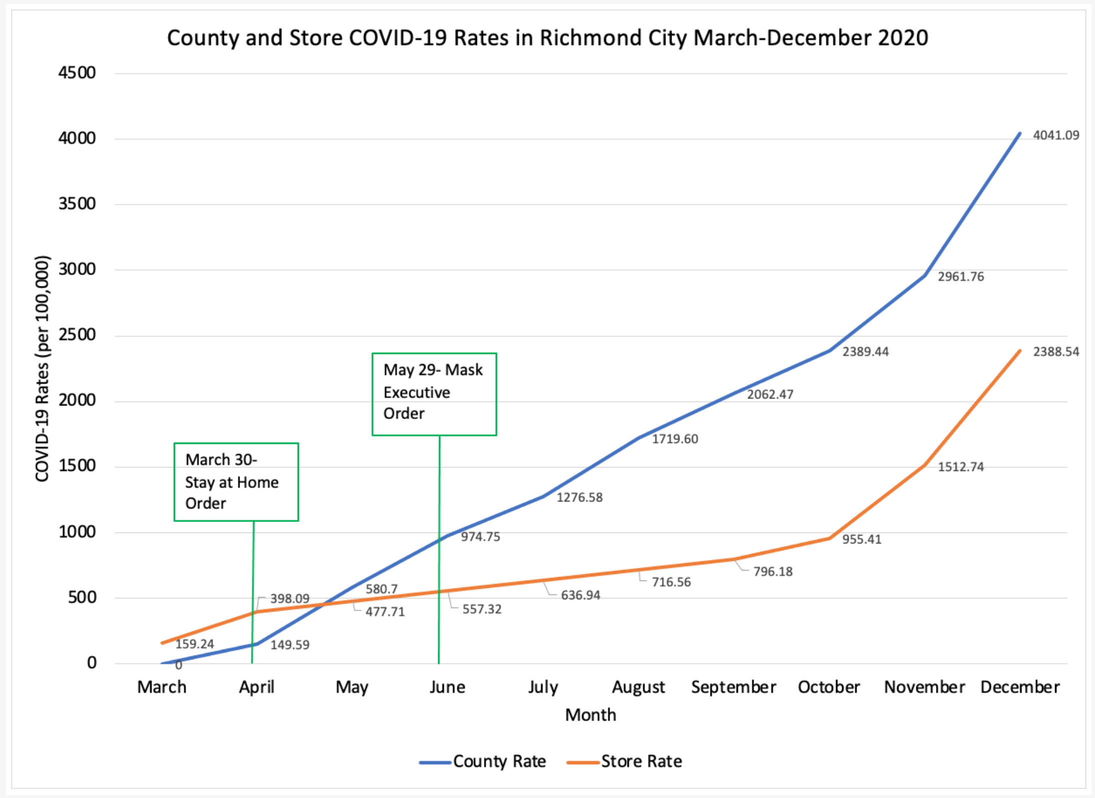

County and Store COVID-19 Rates in Richmond City March-December 2020
COVID-19 Rates (per 100,000)
4500
4000
3500
3000
2500
2000
1500
1000
500
0
March
April
May
June
July
August
September
October
November
December
Month
March 30- Stay at Home Order
May 29- Mask Executive Order
0
149.59
580.7
974.75
1276.58
1719.60
2062.47
2389.44
2961.76
4041.09
159.24
398.09
477.71
557.32
636.94
716.56
796.18
955.41
1512.74
2388.54
County Rate
Store Rate

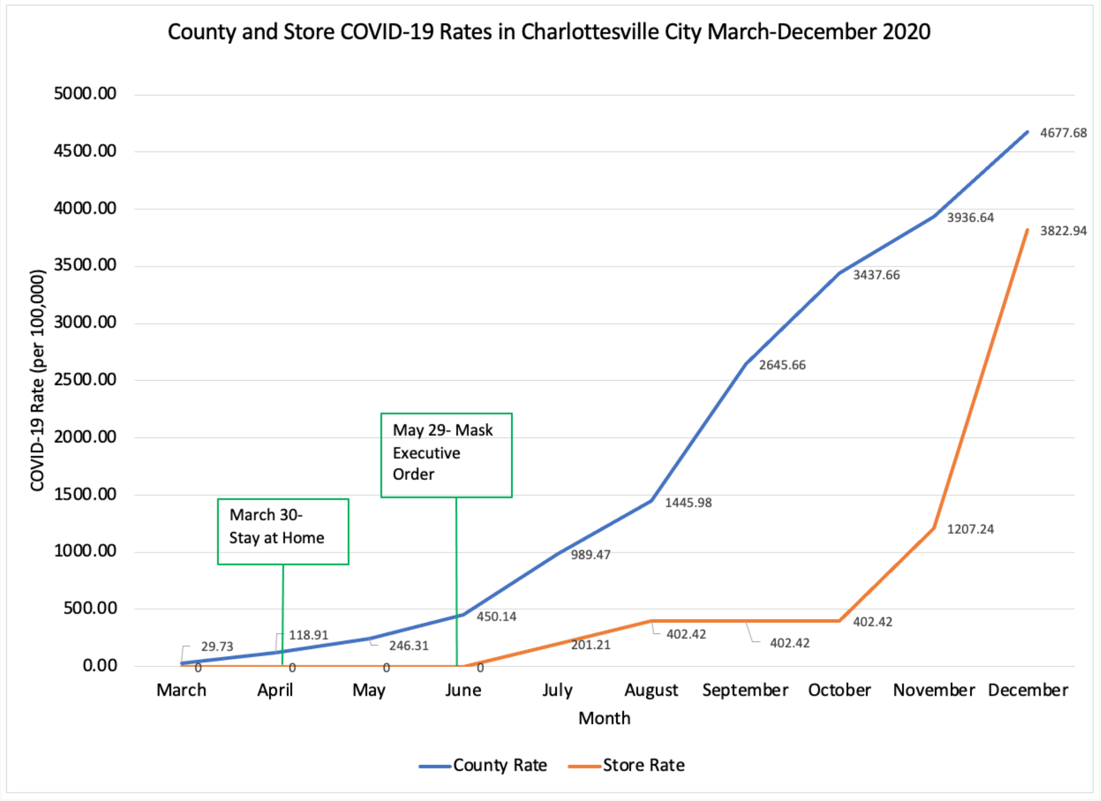

County and Store COVID-19 Rates in Charlottesville City March-December 2020
COVID-19 Rate (per 100,000)
5000.00
4500.00
4000.00
3500.00
3000.00
2500.00
2000.00
1500.00
1000.00
500.00
0.00
March
April
May
June
July
August
September
October
November
December
Month
March 30- Stay at Home
May 29- Mask Executive Order
29.73
118.91
246.31
450.14
989.47
1445.98
2645.66
3437.66
3936.64
4677.68
0
0
0
0
201.21
402.42
402.42
402.42
1207.24
3822.94
County Rate
Store Rate

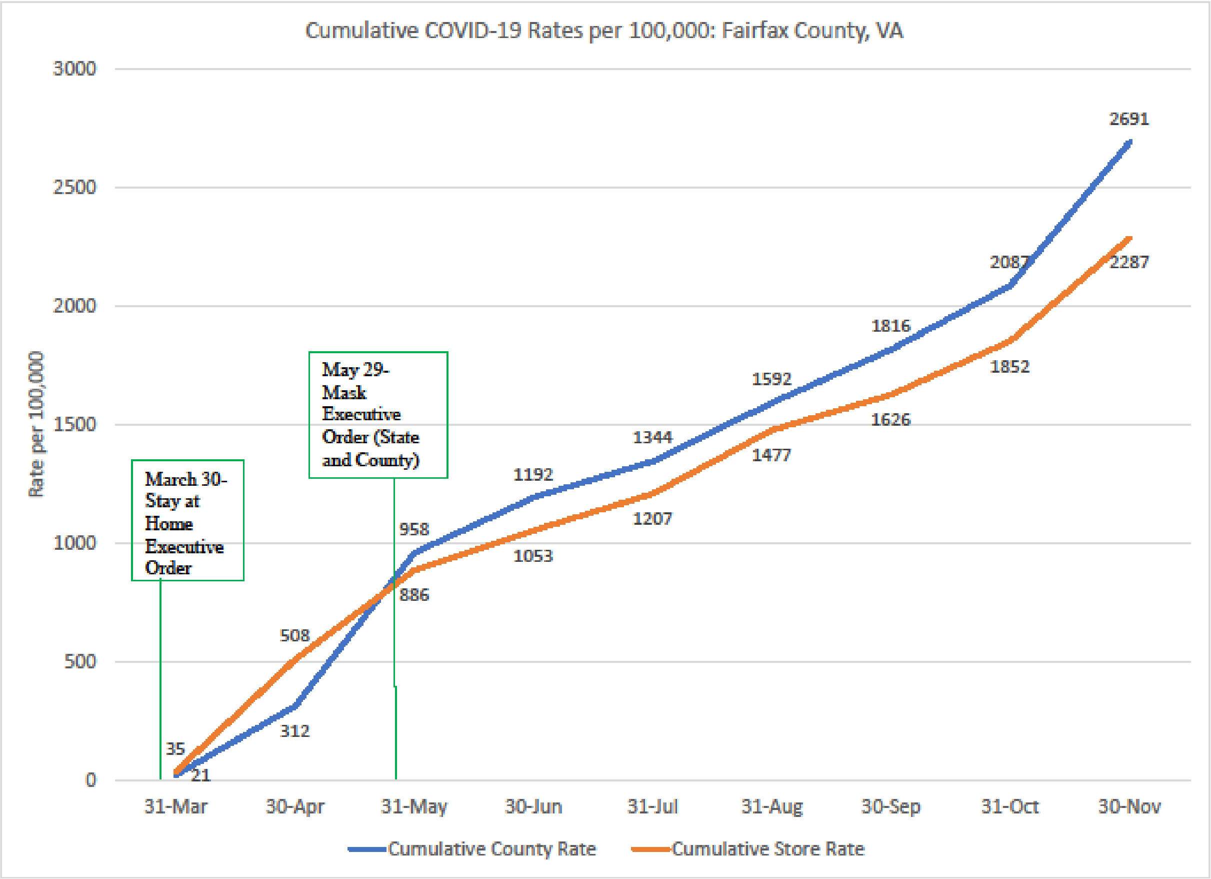

Cumulative COVID-19 Rates per 100,000: Fairfax County, VA
Rate per 100,000
3000
2500
2000
1500
1000
500
0
March 30- Stay at Home Executive Order
May 29- Mask Executive Order (State and County)
35
21
508
312
958
886
1192
1053
1344
1207
1592
1477
1816
1626
2087
1852
2691
2287
31-Mar
30-Apr
31-May
30-Jun
31-Jul
31-Aug
30-Sep
31-Oct
30-Nov
Cumulative County Rate
Cumulative Store Rate

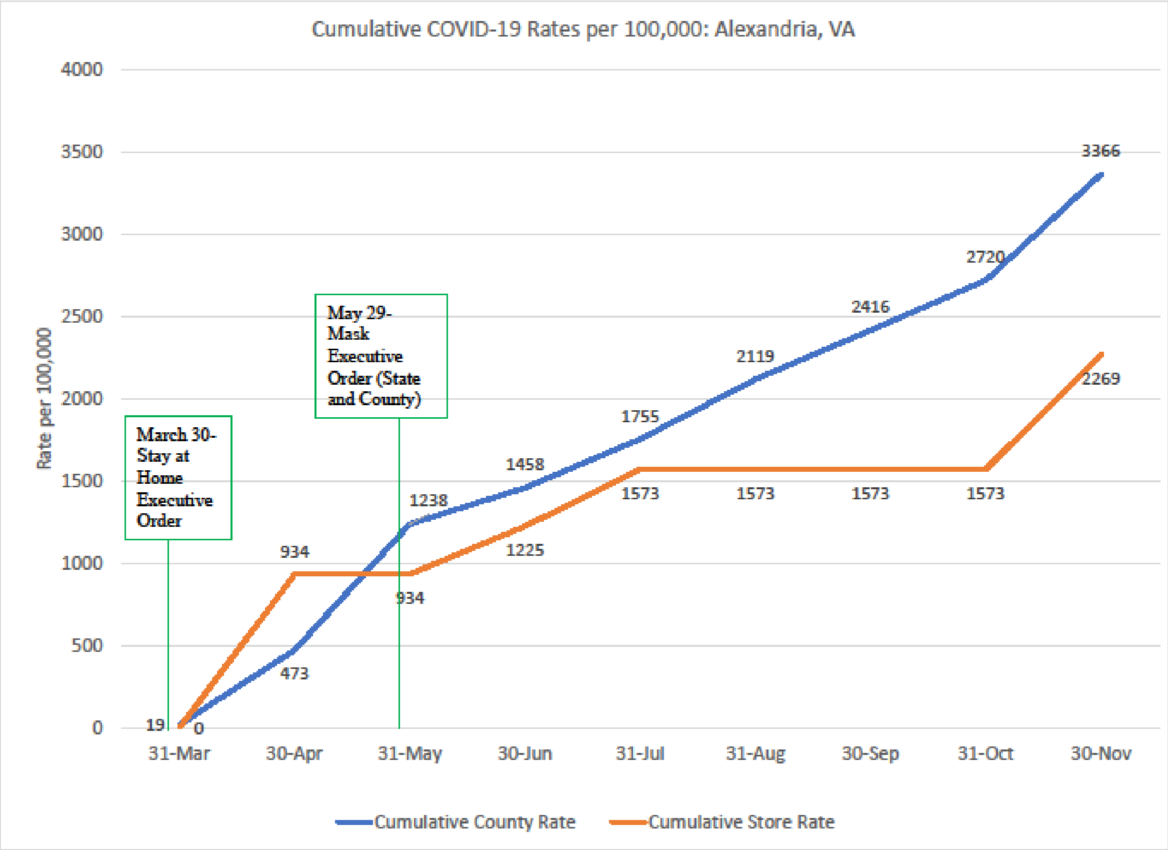

Cumulative COVID-19 Rates per 100,000: Alexandria, VA
Rate per 100,000
4000
3500
3000
2500
2000
1500
1000
500
0
March 30- Stay at Home Executive Order
May 29- Mask Executive Order (State and County)
19
0
934
473
1238
934
1458
1225
1755
1573
2119
1573
2416
1573
2720
1573
3366
2269
31-Mar
30-Apr
31-May
30-Jun
31-Jul
31-Aug
30-Sep
31-Oct
30-Nov
Cumulative County Rate
Cumulative Store Rate

Union Efforts to Reduce Covid-19 Infections among Grocery Store Workers Introduction

Nancy A. Crowell, Alan Hanson, Louisa Boudreau, Robyn Robbins, Rosemary K. Sokas

Supplemental Table

Table 4: Mean Percentage of Time Between April and August 2020 that Stores Practiced Specific Safety Behaviors by Whether or Not Store Covid-19 Rate was Above County Rate or Not

	September 2020			October 2020			November 2020		
Above County Rate:	Above	Below		Above	Below		Above	Below	
	%	%	*P (d) FPR*	%	%	*P (d) FPR*	%	%	*P (d) FPR*
Social Distancing being Practiced	82.9	89.4	.04 (.25) .20	85.1	88.6	.27 (.13) .52	84.4	89.3	.11 (.19) .35
Customer Shopping Limits	71.1	71.0	.99 (-.002) .67	71.1	71.0	.99 (-.001) .67	70.4	71.4	.79 (.03) .66
One Entrance Closed or Used for Exit Only	72.5	70.1	.55 (-.07) .63	70.4	71.0	.86 (.02) .66	71.2	70.3	.73 (-.04) .65
Social Distancing Floor Signs	95.3	96.4	.55 (.07) .63	96.0	96.1	.96 (.01) .67	95.0	96.7	.32 (.12) .55
One-way Aisles	79.9	74.5	.27 (-.14) .52	79.8	74.4	.26 (-.14) .51	78.9	74.5	.34 (-.11) .56
Aisles or Peripheral Departments Closed for Restocking	17.3	10.1	.02 (-.28) .14	13.1	11.8	.67 (-.05) .65	11.6	12.6	.74 (.04) .65
Customers Required to Wear Masks	67.2	71.1	.35 (.12) .56	63.5	72.9	.02 (.28) .11	66.4	72.0	.15 (.17) .41
Management Enforcing Customer Mask Wearing	45.4	53.2	.14 (.18) .40	42.4	54.8	.02 (.29) .10	45.9	53.8	.11 (.19) .36

Above County Rate:	September 2020			October 2020			November 2020		
	Above	Below		Above	Below		Above	Below	
	%	%	*P* *(d)* *FPR*	%	%	*P* *(d)* *FPR*	%	%	*P* *(d)* *FPR*
Hand Washing Every 30 Minutes	73.0	74.9	.67 (.05) .65	75.5	73.8	.69 (-.05) .65	76.0	73.4	.54 (-.07) .62
Hand Sanitizer Available at Work Stations	97.5	98.3	.41 (.10) .59	98.0	98.2	.82 (.03) .66	98.1	98.1	.95 (.01) .67
Work Stations Cleaned Every 30 Minutes	82.6	88.2	.08 (.22) .29	84.0	87.8	.21 (.15) .48	84.2	88.0	.22 (.14) .48
Breakroom Social Distancing	72.2	72.6	.94 (.01) .67	69.9	73.6	.38 (.11) .58	72.3	72.6	.95 (.01) ..67
Employees Required to Wear Masks	97.0	98.0	.48 (.09) .61	97.2	98.0	.61 (.06) .64	97.3	98.0	.61 (.06) .64
Masks/Gloves/ Sanitizer (PPE) Provided by Company	98.2	99.5	.09 (.21) .33	98.3	99.4	.13 (.18) .39	98.6	99.4	.23 (.14) .49
Frequently Touched Surfaces Cleaned Regularly	84.9	90.2	.07 (.23) .27	85.5	90.2	.09 (.20) .32	86.5	90.0	.20 (.15) .47
Training on Proper Putting on/Taking Off of Masks	64.3	66.4	.67 (.05) .65	66.3	65.6	.90 (-.02) .67	64.5	66.6	.65 (.05) .64
Operating Every Other Check Stand	65.7	69.2	.39 (.11) .72	65.8	69.3	.38 (.11) .58	66.8	68.9	.58 (.07) .63
Social Distancing While Waiting in Line	93.9	95.6	.24 (.15) .50	94.1	95.6	.29 (.13) .53	94.1	95.7	.26 (.13) .51
Self Service Checkouts Closed or Operating	44.7	43.6	.80 (-.03) .66	45.8	43.1	.54 (-.03) .64	44.8	43.4	.74 (-.07) .66

Above County Rate:	September 2020			October 2020			November 2020		
	Above	Below		Above	Below		Above	Below	
	%	*%*	*P* *(d)* *FPR*	*%*	*%*	*P* *(d)* *FPR*	*%*	*%*	*P* *(d)* *FPR*
Every Other Station Reusable Bags Banned or Cashiers/Baggers Not Required to Handle	93.4	96.1	.08 (.22) .29	92.4	96.7	.004 (.35) .03	92.7	96.8	.005 (.34) .04

Note: *P*-values are based on independent samples *t* tests; *d* is Cohen's *d*, the standardized effect size (negative values mean percent in stores with above county rate were higher than other stores); *FPR* is False Positive Risk, that is the probability that the results are due solely to chance, calculated using Longstaff and Colquhoun (no date) online calculator. [8]
